# Type 1 and 2 diabetes mellitus: comprehensive fracture risk relationships in UK Biobank

**DOI:** 10.1093/jbmr/zjaf094

**Published:** 2025-07-11

**Authors:** Elizabeth M Curtis, Rebecca J Moon, Stefania D’Angelo, Zahra Raisi-Estabragh, Cyrus Cooper, Nicholas C Harvey

**Affiliations:** MRC Lifecourse Epidemiology Centre, University of Southampton, Southampton General Hospital, Southampton SO16 6YD, United Kingdom; NIHR Southampton Biomedical Research Centre, University of Southampton and University Hospital NHS Foundation Trust, Southampton SO16 6YD, United Kingdom; MRC Lifecourse Epidemiology Centre, University of Southampton, Southampton General Hospital, Southampton SO16 6YD, United Kingdom; Department of Paediatric Endocrinology, University Hospital Southampton NHS Foundation Trust, Southampton SO16 6YD, United Kingdom; MRC Lifecourse Epidemiology Centre, University of Southampton, Southampton General Hospital, Southampton SO16 6YD, United Kingdom; Barts Heart Centre, St Bartholomew’s Hospital, Barts Health NHS Trust, London EC1A 7BE, United Kingdom; William Harvey Research Institute, NIHR Barts Biomedical Research Centre, Queen Mary University of London, London EC1M 6BQ, United Kingdom; MRC Lifecourse Epidemiology Centre, University of Southampton, Southampton General Hospital, Southampton SO16 6YD, United Kingdom; NIHR Southampton Biomedical Research Centre, University of Southampton and University Hospital NHS Foundation Trust, Southampton SO16 6YD, United Kingdom; NIHR Oxford Biomedical Research Centre, University of Oxford, Oxford OX3 9DU, United Kingdom; MRC Lifecourse Epidemiology Centre, University of Southampton, Southampton General Hospital, Southampton SO16 6YD, United Kingdom; NIHR Southampton Biomedical Research Centre, University of Southampton and University Hospital NHS Foundation Trust, Southampton SO16 6YD, United Kingdom

**Keywords:** osteoporosis, epidemiology, type 1 diabetes mellitus, type 2 diabetes mellitus, fracture

## Abstract

We aimed to investigate associations between diabetes mellitus and incident fracture, stratified by diabetes type (1 or 2), disease duration and microvascular complications of diabetes. This prospective cohort analysis used data from the UK Biobank, a large population-based cohort of participants recruited 2006-2010 at age 40-69 yr. The exposure was type 1 or type 2 diabetes at baseline, with the outcome of first incident osteoporotic fracture. Poisson regression was used to calculate incidence rate ratios (IRRs) for osteoporotic fracture to investigate prospective relationships between diabetes type 1 or 2 and fracture risk independent of traditional clinical risk factors, estimated BMD by heel ultrasound (eBMD), adiposity, and C-reactive protein (CRP). The role of diabetic microvascular complications and associations between diabetes duration and fracture risk were studied. There were 498 949 participants (271 882 women, mean age 56 yr; 227 067 men, 57 yr). In fully adjusted models, type 1 and 2 diabetes were associated with increased fracture risk [type 1; IRR: 2.93 (95% CI: 2.37, 3.62); type 2: 1.25 (1.14, 1.38)], similar by sex. The magnitude of risk associated with type 2 diabetes increased with the duration of disease. Increasing number of microvascular complications was associated with greater fracture risk [any vs no complications, IRR 2.03 (1.57, 2.62)]. Diabetes is associated with increased risk of fracture (magnitude of effect greater in type 1 than type 2 diabetes). Associations were partly independent of traditional risk factors, adiposity, eBMD, and CRP. Type 2 diabetes disease duration and the presence of microvascular complications in both types were dose-dependent risk factors for fracture.

## Background

Osteoporosis causes substantial morbidity globally due to fragility fractures and consequent associated mortality and healthcare costs.^1^ Increased fracture risk in both type 1 and type 2 diabetes has been demonstrated across a range of cohorts, despite differences in the underlying pathogenesis.^2^ In general, patients with type 1 diabetes appear to have greater fracture risk than those with type 2, with some studies suggesting no excess fracture risk in the latter.^3–6^ However, the role of BMD and adiposity,^7–9^ chronic low-grade inflammation^2^^,^^10–13^ and microvascular complications^14^^,^^15^ have not been clearly delineated. Indeed, to our knowledge, no cohort has permitted an integrated investigation across all such questions in a uniformly phenotyped, sufficiently large population.

With over 500 000 participants, UK Biobank offers a unique opportunity to assess the association between diabetes and fractures with robust statistical power, allowing for comprehensive analyses and subgroup assessments.^16^ In this dataset, diabetes is one of the most prevalent chronic noncommunicable conditions, with around 22 000 cases reported at baseline and a predicted 40 000 incident cases by 2023.

In UK Biobank, we therefore quantified the risk of incident fractures in individuals with type 1 and type 2 diabetes, considering variation by disease duration and diabetic vascular complications (such as retinopathy, neuropathy, and peripheral vascular disease), together with independence from estimated BMD by heel ultrasound (eBMD), adiposity and proxies for chronic systemic inflammation and renal function.

## Materials and methods

### Setting and recruitment

UK Biobank is a longitudinal population study incorporating over half a million participants recruited during 2006-2010 from across the UK. Individuals aged 40-69 yr old were identified through National Health Service (NHS) registers and invited to participate. The baseline assessment, undertaken at regional centers (2006-2010), included detailed review of demographics, lifestyle, medical history, a series of physical measures, and blood sampling. The protocol is publicly available.^17^ Individuals who were unable to consent or complete the baseline assessment due to illness or discomfort were not recruited. Linkage with Hospital Episode Statistics (HES) and death registers enable longitudinal tracking of health outcomes for all participants. This work was undertaken using the UK Biobank resource under approved application no. 3593.

### Ethics

This study is covered by the ethical approval for UK Biobank studies from the NHS National Research Ethics Service on June 17, 2011 (Ref. 11/NW/0382) and renewed June 29, 2021 (Ref. 21/NW/0157).

### Exposure: diabetes mellitus type 1 or type 2

The identification of prevalent diabetes cases has been recognized as a challenge in observational studies, as the validity of diabetes self-report varies considerably.^18^^,^^19^ We used the algorithm devised by Eastwood et al. in UK Biobank to classify individuals as either “Diabetes unlikely,” “type 1,” or “type 2.”^20^ This incorporates a combination of self-reported data, nurse interviews, and linkage to specific ICD10 codes to assign a status of “probable” or “possible” diabetes. On detailed review of the algorithm, it was judged that the classification of the modest number of individuals with “possible” type 2 diabetes was insufficiently robust to be certain that they did not have type 1 diabetes. We therefore included only cases of “probable” type 2 diabetes in the analysis, together with both “probable” and “possible” cases of type 1 diabetes (where it was judged that both classifications were likely to be robust). We removed from the analysis women with “possible gestational diabetes” due to the short duration and differing pathogenesis of this condition (*n* = 192).

We made the following clinically-informed assumptions: for participants whose age at type 1 diabetes diagnosis was >40 yr, we assumed that this variable had been entered as “years since diagnosis” instead of age of onset (*n* = 80). Conversely, for participants whose age at type 2 diabetes diagnosis was <10 yr, we have assumed that this variable was actually “years since diagnosis” (*n* = 214).

### Microvascular complications of diabetes, and comorbidities

We defined complications of diabetes based on ICD10 codes, from linked HES records (and equivalent linkage outside England): G590: diabetic mononeuropathy; G632: diabetic polyneuropathy; H280: diabetic cataract; H360: diabetic retinopathy; N083: glomerular disorders in diabetes mellitus. We combined retinopathy and diabetic cataract into eye complications, and diabetic polyneuropathy and diabetic mononeuropathy into neuropathy, thereby avoiding double counting if a participant had more than one condition in the same category. Comorbidity count used the conditions listed in the Charlson comorbidity index.^21^ In a further analysis, we used estimated glomerular filtration rate (eGFR) as a proxy measure of renal function, likely to be altered by renal microvascular disease. We used the glomerular filtration rate (GFR) estimating equations reported by the National Institute of Diabetes and Digestive and Kidney Diseases, as outlined in Table S10.^22^

### Outcome: osteoporotic fracture

In order to maximize statistical power, we used first incident (after baseline) osteoporotic fracture as the principal outcome, obtained through HES (clinical vertebral, ribs, pelvis, humerus, clavicle, scapula, sternum, hip, other femoral fractures, tibia, fibula, and distal forearm/wrist).^23^ ICD9 and ICD10 codes are recorded in the supplementary material (Table S9). Information on the use of HES data in UK Biobank is given here: https://biobank.ndph.ox.ac.uk/showcase/refer.cgi?id=138483.

### Baseline measures

Heel estimated BMD (eBMD) was derived from quantitative ultrasound (QUS, Hologic Sahara) at the calcaneus bilaterally in UK Biobank participants, according to a predefined standard operating procedure.^24^ Quality control checks of the sonometer were undertaken daily using a phantom. QUS parameters have been shown to be independent predictors of fragility fractures and demonstrate moderate associations with BMD measured by DXA.^25^^,^^26^^,^^27^ Whole body fat mass was measured by bioimpedance analysis (Tanita BC418MA body composition analyzer).^28^ Standing height was measured using a Seca 202 measuring rod. BMI was calculated by dividing body weight by the squared height (kg/m^2^). We considered serum C-reactive protein (CRP) as a surrogate for chronic systemic inflammation, and eGFR as a marker of renal function. Details of the biochemical methods are available on the UK Biobank website (https://www.ukbiobank.ac.uk/media/oiudpjqa/bcm023_ukb_biomarker_panel_website_v1-0-aug-2015-edit-2018.pdf). Information on medications was obtained through self-report and nurse interview at baseline.

### Statistical analysis

Descriptive baseline statistics are presented by diabetes status, using counts and percentages, mean and SD for normally distributed continuous variables and median with IQR for other continuous variables.

We investigated the association between diabetes status and heel eBMD, stratified by sex. To delineate the associations between baseline diabetic status and incident osteoporotic fracture, we used Poisson regression models with robust standard errors, since the proportional hazards assumption was not met. Individuals were followed until the first fracture event, death, withdrawal from the database, or until September 30, 2021. As well as the unadjusted model, we undertook further models adjusted for the following confounders: sex, age, tobacco use, alcohol use, deprivation index score, level of physical activity, co-morbidities (Model 1), Model 1 + heel eBMD, Model 1 + body fat mass, Model 1 + CRP, Model 1 + eGFR, and Model 1 + heel eBMD + body fat mass + CRP + eGFR (Model 2). Assumptions notwithstanding, analysis using Cox proportional hazards models gave very similar results, and we therefore undertook Fine and Gray competing risk models to investigate whether mortality modified the effect sizes.

We investigated potential interactions between diabetes status, age, sex, and BMI performing stratified analysis by category of BMI: underweight <18.5 kg/m^2^; normal weight 18.5-25 kg/m^2^; overweight 25-30 kg/m^2^; obese >30 kg/m^2^. We also performed analyses on diabetic complications and tested the associations by different types of complications (eye complications, neuropathy, and glomerular disorders) and number of complications on osteoporotic fractures. We analyzed the risk of osteoporotic fracture by disease duration (enabling us to compare, e.g., those with disease duration of less than a year compared to more than a year, less than 2 yr compared with more than 2 yr, etc.).

Finally, we undertook sensitivity analyses to investigate the effect of our assumptions by (1) omitting those individuals who were reclassified on the basis of disease duration or age of onset, and (2) including those excluded on the basis of classification as “Possible Type 2 Diabetes.”

Statistical analyses were performed using STATA Version 17.0.

## Results

### Study population

A total of 498 949 UK Biobank participants were included in these analyses, of whom 1836 had type 1 diabetes and 20 551 had type 2 diabetes. Table 1 documents baseline characteristics by diabetes status. The mean baseline age was 58 yr for those with no diabetes, 55 yr with type 1 diabetes, and 62 yr with type 2 diabetes.

**Table 1:** Characteristics of the sample by diabetes status.

**Category**	***N* (%), median (IQR) or mean (SD)**		
	No diabetes (*n* = 476 562)	Type 1 (*n* = 1836)	Type 2 (*n* = 20 551)
**Sex, male**	213 091 (44.7)	991 (54.0)	12 985 (63.2)
**No. of years since diagnosis of diabetes**	-	31 (22-39)	4 (2-8)
**Age, yr**	58 (50-63)	55 (47-61)	62 (56-66)
**Heel eBMD (g/cm^2^)**	0.54 (0.14)	0.53 (0.14)	0.57 (0.16)
**Fat mass (kg)**	24.5 (9.3)	24.7 (10.9)	31.4 (11.8)
**BMI, kg/m^2^**	27.2 (4.6)	28.2 (5.4)	31.7 (5.8)
** Underweight**	2593 (0.6)	4 (0.2)	16 (0.1)
** Normal weight**	159 459 (33.7)	540 (30.1)	1893 (9.3)
** Overweight**	203 298 (42.9)	719 (40.0)	6993 (34.3)
** Obese**	108 389 (22.9)	533 (29.7)	11 471 (56.3)
**Ethnicity**			
** European/other**	461 879 (97.1)	1775 (96.7)	18 624 (90.9)
** South Asian/African Caribbean**	13 849 (2.9)	61 (3.3)	1870 (9.1)
**Smoking status**			
**Never**	261 796 (55.2)	1009 (55.3)	9141 (45.0)
**Previous**	161 971 (34.2)	599 (32.8)	8991 (44.2)
**Current**	20 116 (10.6)	217 (11.9)	2197 (10.8)
**Alcohol consumption**			
**Daily or almost daily**	98 051 (20.6)	342 (18.7)	2942 (14.4)
**Three or four times a week**	111 493 (23.5)	333 (18.2)	3168 (15.5)
**Once or twice a week**	123 359 (26.0)	438 (23.9)	4682 (22.9)
**One to three times a month**	52 715 (11.1)	190 (10.4)	2519 (12.3)
**Special occasions only**	53 168 (11.2)	272 (14.9)	3872 (18.9)
**Never**	36 396 (7.7)	257 (14.0)	3271 (16.0)
**Level of education**			
**College/University**	155 157 (33.2)	579 (32.2)	4617 (23.2)
**Other professional qualification/HDH/A levels**	154 519 (33.1)	590 (32.8)	6486 (32.6)
**GCSE or less**	157 522 (33.7)	632 (35.1)	8807 (44.2)
**No. of comorbidities**			
**0**	369 280 (77.5)	796 (43.4)	12 033 (58.6)
**1**	35 217 (7.4)	356 (19.4)	3150 (15.3)
**2+**	72 065 (15.1)	684 (37.3)	5368 (26.1)
**Has diabetic complications**	-	807 (44.0)	2410 (11.7)
**Number of complications**	-		
** 1**		510 (63.2)	2025 (84.0)
** 2+**		297 (36.8)	385 (16.0)
**C-reactive protein, mg/L**	1.3 (0.7, 2.7)	1.6 (0.8, 3.6)	1.8 (0.9, 3.8)
**Estimated glomerular filtration rate, mL/min/1.73 m^2^**	94.8 (13.0)	94.3 (20.1)	92.6 (15.7)
**No. of individuals with incident osteoporotic fracture**	23 925 (5.0)	219 (11.9)	1271 (6.2)
**Osteoporotic fracture incidence, /1000 person-years**	4.1 (4.0, 4.1)	10.0 (8.8, 11.4)	5.0 (4.8, 5.3)
**No. of deaths**	32 974 (6.9)	368 (20.0)	3675 (17.9)

Participants with type 2 diabetes were older, more likely to be male, obese, of South Asian or African Caribbean ethnicity, and with lower levels of college/university education compared with the population without diabetes (Table 1**)**. Comorbidities were more common in individuals with diabetes than those without, and these were more common in those with type 1 diabetes (individuals with 2 or more comorbidities, 26.1% of those with type 2 diabetes, 37.3% of those with type 1 diabetes, and 15.1% of those without diabetes). Consistently, the number of diabetic complications was also greater in the type 1 diabetes group (36.8% having two or more diabetic complications, compared with 16% in the type 2 diabetes group). A greater number of deaths during follow-up were observed in participants with diabetes (20% type 1 diabetes and 17.9% type 2 diabetes), compared with those without diabetes (6.9%). In participants without diabetes, 5% suffered an incident osteoporotic fracture, compared with 11.9% in individuals with type 1 diabetes and 6.2% in those with type 2 diabetes.

### Associations of type 1 or type 2 diabetes with heel eBMD

Table 2 illustrates the relationships between type 1 or type 2 diabetes and heel eBMD. Compared with participants without diabetes, type 1 diabetes was associated with lower heel eBMD, whereas type 2 diabetes was associated with higher heel eBMD. These associations were robust to adjustment for fat mass measured by bioimpedance. The magnitude of the associations differed by sex and diabetes type: type 1 diabetes was negatively associated with heel eBMD, more strongly in men than women [after adjustment for fat mass, in men, β = −0.03 (95% CI: −0.04, −0.02) g/cm^2^; in women, β = −0.006 (−0.0148, 0.002) g/cm^2^]. The presence of type 2 diabetes was positively associated with heel eBMD in both women and men, with stronger associations observed in women [(after adjustment for fat mass, in men, β = 0.006 (95% CI: 0.003, 0.008) g/cm^2^; in women, β = 0.015 (0.012, 0.018) g/cm^2^].

**Table 2:** Association between type 1 and type 2 diabetes vs no diabetes and heel BMD (g/cm^2^) by sex.

	**Women**		**Men**	
	*N*	β (95% CI) g/cm^2^	*N*	β (95% CI) g/cm^2^
**Type 1 diabetes**				
** Unadjusted**	256 170	−0.005 (−0.013, 0.003)	206 245	−0.028 (−0.038, -0.019)
** Adjusted for fat mass**	252 984	−0.006 (−0.148, 0.002)	202 645	−0.030 (−0.040, -0.021)
**Type 2 diabetes**				
** Unadjusted**	262 584	0.024 (0.021, 0.027)	217 663	0.015 (0.012, 0.017)
** Adjusted for fat mass**	259 287	0.015 (0.012, 0.018)	231 785	0.006 (0.003, 0.008)

Unadjusted associations and associations adjusted for fat mass by bioimpedance.

β coefficient represents mean difference in heel BMD between each pair of groups.

### Diabetes and risk of incident fracture

**Figure 1:**
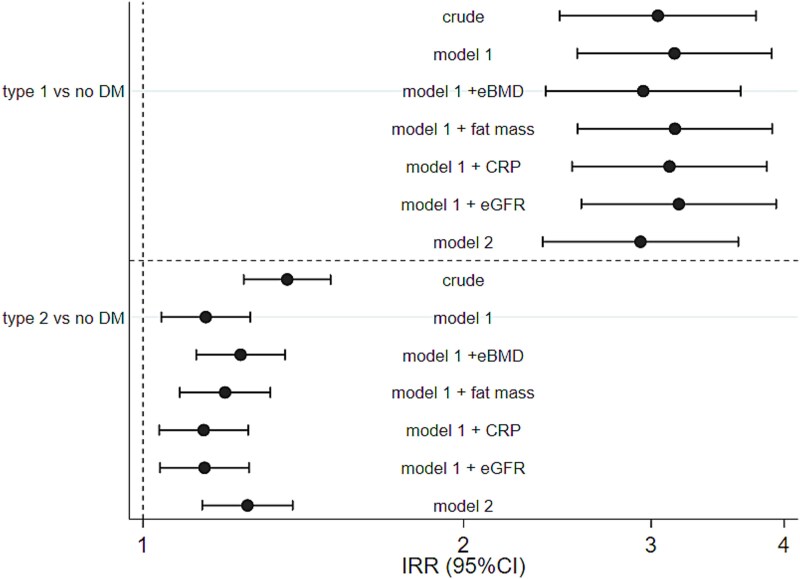
Diabetes and risk of incident osteoporotic fracture. Model 1, covariates: sex, age, tobacco use, alcohol use, deprivation index score, level of physical activity, co-morbidities; model 2 = model 1 + eBMD, fat mass, CRP and eGFR. Risk estimates are derived from a Poisson regression model, with robust standard errors.

Figure 1 and Table S1 illustrate that participants with type 1 diabetes were approximately 3 times more likely than those without diabetes to experience an incident osteoporotic fracture (Model 1; incidence rate ratio [IRR] 3.16 (2.56, 3.89), *p* < 0.001). Full adjustment (Model 2) incorporating eBMD, fat mass, CRP, and eGFR did not materially alter the observed association (Model 2: IRR 2.93 (2.37, 3.62), *p* < 0.001). Type 2 diabetes was also associated with increased fracture risk albeit with the quantum of effect notably smaller than that with type 1 diabetes. (Model 1: IRR 1.15 (1.04, 1.26), *p* = 0.006). Full adjustment incorporating eBMD, fat mass, CRP, and eGFR again did not materially alter the observed association (Model 2: IRR 1.25 (1.14, 1.38), *p* < 0.001).

In a Fine and Gray competing risk model, adjustment for death hazard did not materially alter the findings but did attenuate the magnitude of the effect of type 1 diabetes on fracture risk; type 1 diabetes Model 1: sub-distribution hazard ratio (SHR) 2.46 (2.10, 2.88); and type 2 diabetes Model 1: SHR 1.18 (1.11, 1.27).

**Figure 2:**
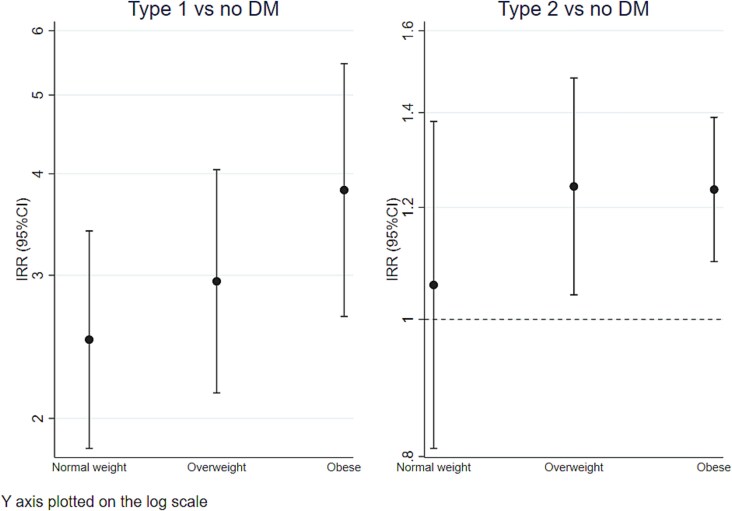
Type 1 and type 2 diabetes and osteoporotic fracture risk, by BMI category: normal weight (18.5-25), overweight (25-30), and obese (>30 kg/m^2^). Note that Y-axis is a logarithmic scale. Model 1, covariates: sex, age, tobacco use, alcohol use, deprivation index score, level of physical activity, and co-morbidities.

There was no evidence of an interaction with either sex or age in the association between incident osteoporotic fracture and diabetes of either type. However, we observed evidence of an interaction between type 2 diabetes (but not type 1 diabetes) and BMI (Table S2, *p* interaction = 0.03). Therefore, we performed analysis stratified by BMI (Figure 2 and Table S3). In individuals with type 1 diabetes the risk of osteoporotic fracture was raised irrespective of BMI, although there was a trend with increasing risk with increasing BMI category. The association between type 2 diabetes and fractures was however only significant for participants in the overweight or obese categories with similar IRRs of around 1.2. The underweight category is not shown in the figures as the model did not converge due to a very small number of participants in this category.

### Associations between microvascular complications of diabetes and incident fractures

Table 3 is restricted to participants with diabetes and documents that, although any type of microvascular complication was associated with increased risk of fracture, risks were particularly elevated for participants with neuropathy and glomerular disorders, which increased risks of fracture by 2- or 3-fold, irrespective of the type of diabetes. We detected a dose-response effect of number of complications on fracture risk (Table S4), with risks more than trebling for participants with diabetes (either type 1 or type 2) and at least 2 complications vs those with diabetes and without complications (IRR = 3.69; 95% CI: 2.78, 4.91), compared with a doubling for 1 vs no complications (IRR 1.97; 95% CI: 1.57, 2.47). The risk of complications was strongly associated with BMI, for example, in participants with eye complications, 51% (*n* = 1278) were living with obesity compared to 13% with normal BMI (*n* = 327), and for neuropathies, 57% (*n* = 532) were living with obesity compared to 12% (*n* = 111) with normal BMI (Table S5).

**Table 3:** Association between microvascular complications of diabetes and incident fracture in comparison to diabetes without complications, stratified by type of diabetes.

	**Diabetes with any microvascular complication**		**Diabetes with eye complications**		**Diabetes with neuropathy**		**Diabetes with glomerular disorders**	
	*n*	IRR (95% CI)	*n*	IRR (95% CI)	*n*	IRR (95% CI)	*n*	IRR (95% CI)
**Type 1 DM**								
** Osteoporotic fracture**								
** Unadjusted**	1836	2.28 (1.55, 3.33)	1767	2.23 (1.51, 3.30)	1314	3.01 (1.91, 4.73)	1836	2.28 (1.41, 3.67)
** Model 1**	1662	1.65 (1.11, 2.46)	1604	1.57 (1.05, 2.35)	1188	2.28 (1.35, 3.85)	1662	2.37 (1.41, 3.98)
** Model 2**	1408	1.52 (1.00, 2.29)	1363	1.41(0.93, 2.15)	1000	2.15 (1.25, 3.71)	1408	1.86 (0.97, 3.59)
**Type 2 DM**								
** Osteoporotic fracture**								
** Unadjusted**	20 551	2.28 (1.82, 2.87)	19 959	1.84 (1.42, 2.39)	18 816	3.77 (2.65, 5.36)	20 551	3.53 (2.19, 5.68)
** Model 1**	18 092	2.04 (1.59, 2.61)	17 590	1.84 (1.38, 2.45)	16 604	3.15 (2.18, 4.55)	18 092	2.23 (1.44, 3.46)
** Model 2**	15 960	2.05 (1.59, 2.64)	15 522	1.76 (1.33, 2.32)	14 668	3.27 (2.16, 4.94)	15 960	2.72 (1.68, 4.40)

Diabetic complications: any of diabetic mononeuropathy, polyneuropathy, cataract, retinopathy, arthropathy, and glomerular disorders.

Model 1 covariates: sex, age, tobacco use, alcohol use, deprivation index score, level of physical activity, and co-morbidities; model 2: model 1 + eBMD, fat mass and CRP, and eGFR.

### Diabetes duration

Table S6 documents the time-specific analyses for relationships between duration of type 2 diabetes and risk of osteoporotic fracture, year on year. The incident rate ratio for fracture became positive at around 5 yr duration, being lower than unity for disease duration less than 5 yr. Thus, the association between diabetes type 2 and osteoporotic fracture was close to unity for disease duration up to 5 yr [1.02 (0.89, 1.17)], but with a 28% increase in risk for duration beyond 5 yr [1.28 (1.13, 1.44)].

### Sensitivity analyses

Table S7 demonstrates our reclassification approach had no material impact on the associations. Since a proportion of the “possible type 2 diabetes” group will be type I diabetes, the greater magnitude of effect size in the relationship between diabetes and fracture, apparent when including these participants, is consistent with this assumption (Table S8).

## Discussion

In this study using UK Biobank, we demonstrated that both type 1 and type 2 diabetes are associated with an increased risk of osteoporotic fractures, with the quantum of effect being greater for type 1 than for type 2. There were effects of duration of disease for type 2, and from the presence/absence of diabetic microvascular complications for both diabetes types on fracture risk. These associations were not materially modified by consideration of adiposity, heel eBMD, CRP, or eGFR. To our knowledge, this is the largest investigation of the associations between diabetes and fracture risk in a single uniformly deeply phenotyped cohort with consistently validated outcome ascertainment.

The increased frequency of osteoporotic fractures in individuals with type 1 and type 2 diabetes compared with those without diabetes is consistent with previous literature,^3^^,^^4^^,^^29^ though, converse to our findings, some studies have suggested that type 2 diabetes is not a clinically relevant risk factor for fracture.^30^ We hypothesize that such null findings reflect shorter duration of disease, given that we observed disease duration-dependent associations between type 2 diabetes and incident fracture risk. Indeed, similarly, in a Canadian study of over 82 000 patients with diabetes (type 1 and type 2 combined), those newly diagnosed had lower fracture risk, and those with a longer duration a higher risk, for osteoporotic fracture compared with controls without diabetes.^31^ Our documentation of greater fracture risk with increasing numbers of microvascular complications is also consistent with a dose-response effect in terms of disease severity and duration.

Other studies have identified opposing associations between type 1 and type 2 diabetes with BMD, with the presence of type 1 diabetes being associated with lower BMD, particularly at the hip,^32^ and type 2 diabetes being associated with greater BMD,^8^^,^^33^ with BMI as a major determinant of BMD at both the spine and hip.^34^ The relationships between diabetes, BMI, BMD, and fracture risk are complex. In UK Biobank, we found that in patients with type 1 diabetes, fracture risk was elevated regardless of BMI, with a trend toward greater risk in overweight and obese individuals. In those with type 2 diabetes however, we observed no elevation in fracture risk in normal weight individuals but similarly increased risks in overweight and obese individuals. Interestingly, in a nationwide study of patients with diabetes and controls from Scotland, risk of hip fracture was only slightly increased in patients with type 2 diabetes. The risk was dependent on BMI, in that patients with type 2 diabetes and a low BMI had increased risk and those with higher BMI had lower risk.^35^ In an Italian study (with type 1 and type 2 diabetes combined), consistent with our findings, obese patients with diabetes had higher BMD values than patients with diabetes who were not obese and individuals without diabetes who were obese, but, despite this difference, they still had a greater rate of non-vertebral-non-hip fractures.^36^ These associations (of increased risks of fracture in diabetes with obesity) were not observed in hip and vertebral fractures, with potential mechanical reasons relating to the types of falls and cushioning effects of fat influencing these trends.^37^

The mechanisms linking diabetes and increased fracture risk are believed to be complex and multifactorial. They include increased bone resorption as a consequence of elevated levels of osteoclast stimulation due to the presence of inflammatory cytokines, such as interleukin 1 and TNF-alpha.^2^ In addition, chronic hyperglycemia leads to the accumulation of advanced-glycation end products (AGEs), which impair osteoblast function. Micro- and macrovascular complications, partly due to greater vascular calcification, impair blood flow to bone tissue, and may compromise bone healing.^2^ Diabetes also has other sequelae, including renal impairment (which is also partly due to microvascular disease) and consequent renal bone disease. We were able to detect an association between eGFR and incident osteoporotic fractures, and found a small, but significant, effect size indicating a protective effect of better renal function on fracture (IRR = 0.99 (95% CI: 0.98-0.99)). Diabetes-related complications can also affect falls risk (indeed we observed a particular increase in fracture risk associated with peripheral neuropathy in diabetes), as can hypoglycemic episodes.

These mechanistic considerations are likely to be of relevance to our findings. For example, the greater adiposity associated with type 2 diabetes is likely to be protective for many (but not all) fracture types, via greater BMD, and potentially through biomechanical protection.^38^ Insulin is anabolic to bone and hyperinsulinemia in type 2 diabetes may offset bone fragility resulting from hyperglycemia in early disease, with the negative effect predominating with longer duration.^2^ Although we did not observe any effect modification by adjustment for CRP, this is only one marker of chronic inflammation.^10^ Such nuances may further explain some of the heterogeneity between studies noted above.

### Strengths and limitations

Several limitations should be considered as follows. First, as with many such cohorts, there is evidence of healthy selection bias in UK Biobank compared with the UK population. This may reduce the generalizability of our findings, but notwithstanding, a clinically meaningful distribution of exposures and outcomes was available. Second, it was not possible to undertake meaningful analyses stratified by ethnicity. Third, it is possible that more minor fractures not leading to hospital admission are under-ascertained in the HES data through the use of ICD9 and ICD10 codes; if anything though this would conservatively bias effect estimates. Fourth, while we were able to account for a range of confounders, the potential for residual confounding remains, and we cannot infer causality in this setting. Finally, we were not able to use time to event analysis since the proportional hazards assumptions for Cox Regression were not met. However, the findings from Cox regression were similar to those from Poisson regression.

### Clinical implications

Type 1 diabetes is one of the “secondary osteoporosis” options as an input variable into the current FRAX® calculator, an integral component of over 100 guidelines internationally.^39^ Although the quantum of the risk relationship with incident osteoporotic fracture is substantially greater for type 1 than type 2 diabetes, our findings confirm those from previous small individual studies and meta-analyses, that type 2 diabetes influences fracture risk independent of clinical risk factors, adiposity and BMD. As such, albeit in a rather complex milieu of influences across body composition, skeletal health and inflammatory processes, our findings clearly support consideration of type 2 diabetes in fracture risk assessment, irrespective of the underlying causal mechanisms. Furthermore, while femoral neck BMD from DXA is the potential input variable for FRAX rather than heel ultrasound eBMD, it is notable that the increased risk of fracture with type 1 diabetes persisted after eBMD adjustment. As a “secondary osteoporosis” risk factor, the effect of type 1 diabetes on fracture risk in FRAX is assumed to be via BMD. Our findings suggest that type 1 diabetes might increase fracture risk beyond what is explained by BMD. Importantly, FRAX2 is under development, and will consider inclusion of type 1 and type 2 diabetes, reflecting BMD dependent and independent effects.^40^

## Conclusions

In conclusion, we have quantified, in the uniquely large and well phenotyped UK Biobank population, the increased fracture risk associated with type 1 or type 2 diabetes. The magnitude of effect appears greater in type 1 than type 2 diabetes, and is at least partly independent of eBMD, adiposity and proxies for renal function and chronic inflammation. Importantly, fracture risk increases with type 2 diabetes disease duration, and is markedly increased with microvascular complications in both diabetes types. Our findings are consistent with the known biology of these conditions, and directly inform approaches to clinical risk assessment, in which skeletal health should form part of overall assessment of all patients with diabetes.

## Supplementary Material

Supplementary_tables_DM_fracture_UKB_JBMR_2025_07_06_R1_zjaf094

## Data Availability

All data are available via an access application to UK Biobank (https://www.ukbiobank.ac.uk/enable-your-research).
