# Supplementary material for: Type 1 and 2 diabetes mellitus: comprehensive fracture risk relationships in UK Biobank
Source: J Bone Miner Res. 2025 Jul 11;40(11):1246–54. doi: 10.1093/jbmr/zjaf094 (PMC12578282; doi:10.1093/jbmr/zjaf094)
Supplement: Supplementary_tables_DM_fracture_UKB_JBMR_2025_07_06_R1_zjaf094 [file supplementary_tables_dm_fracture_ukb_jbmr_2025_07_06_r1_zjaf094.docx]

**Supplementary Material**

**Fracture risk in type 1 and 2 diabetes mellitus: effects of duration and microvascular complications in UK Biobank**

Elizabeth M Curtis, PhD^1,2^ Rebecca J Moon PhD^1,3^, Stefania D’Angelo MSci^1^, Zahra Raisi-Estabragh, PhD^4,5^, Cyrus Cooper, FMedSci^1,2,6^, Nicholas C Harvey, PhD^1,2^

1.MRC Lifecourse Epidemiology Centre, University of Southampton, Southampton General Hospital, Southampton UK

2.NIHR Southampton Biomedical Research Centre, University of Southampton and University Hospital NHS Foundation Trust, Southampton, UK

3. Department of Paediatric Endocrinology, University Hospital Southampton NHS Foundation Trust, Southampton, UK

4. Barts Heart Centre, St Bartholomew’s Hospital, Barts Health NHS Trust, West Smithfield, London, UK

5. William Harvey Research Institute, NIHR Barts Biomedical Research Centre, Queen Mary University of London, Charterhouse Square, London, EC1M 6BQ, UK

6. NIHR Oxford Biomedical Research Centre, University of Oxford, UK

**Supplementary Table 1: Associations between Type 1 and Type 2 diabetes and incident fractures**

|  | Type 1 diabetes vs no diabetes | | | Type 2 diabetes vs no diabetes | | |
| --- | --- | --- | --- | --- | --- | --- |
|  | n | IRR (95%CI) | p | n | IRR (95%CI) | p |
| **Incident osteoporotic fracture** | | |  |  |  |  |
| *Unadjusted* | 402833 | 3.04 (2.46,3.76) | <0.001 | 417385 | 1.37 (1.24,1.50) | <0.001 |
| *Model 1* | 402833 | 3.16 (2.56,3.89) | <0.001 | 417385 | 1.15 (1.04,1.26) | 0.006 |
| *Model 1+eBMD* | 402833 | 2.95 (2.39,3.64) | <0.001 | 417385 | 1.24 (1.12,1.36) | <0.001 |
| *Model 1+fat mass* | 402833 | 3.16 (2.56,3.90) | <0.001 | 417385 | 1.19 (1.09,1.32) | <0.001 |
| *Model 1+CRP* | 402833 | 3.12 (2.53,3.85) | <0.001 | 417385 | 1.14 (1.04,1.26) | 0.007 |
| *Model 1+eGFR* | 402833 | 3.19 (2.58,3.93) | <0.001 | 417385 | 1.14 (1.04,1.26) | 0.007 |
| *Model 2* | 402833 | 2.93 (2.37,3.62) | <0.001 | 417385 | 1.25 (1.14,1.38) | <0.001 |

Model 1, covariates: sex, age, tobacco use, alcohol use, deprivation index score, level of physical activity, co-morbidities; model 2 = model 1+ eBMD, fat mass and CRP, and eGFR

Risk estimates are derived from a Poisson regression model, with robust standard errors

| ***Incident osteoporotic fracture*** | **Type 1 diabetes vs**  **no diabetes** | | | **Type 2 diabetes vs**  **no diabetes** | |
| --- | --- | --- | --- | --- | --- |
|  | **n** | **p interaction** | | **n** | **p interaction** |
| ***Age*** | 444599 | | 0.44 | 461029 | 0.67 |
| ***Sex*** | 444599 | | 0.97 | 461029 | 0.09 |
| ***BMI*** | 442548 | | 0.12 | 458877 | 0.03 |

**Supplementary Table 2: Interaction between type 1 or type 2 diabetes versus no diabetes and incident osteoporotic fracture: age, sex, and BMI**

**Supplementary Table 3: Association between diabetes and osteoporotic fracture, by BMI categories**

Model 1, covariates: sex, age, tobacco use, alcohol use, deprivation index score, level of physical activity, co-morbidities

The model did not converge in the Type 1 DM underweight category due to the small number of individuals in this group.

|  | <18.5 kg/m^2^ (Underweight) | | | | 18.5-25 kg/m^2^ (Normal) | | | 25-30 kg/m^2^ (Overweight) | | | | | >30 kg/m^2^ (Obese) | | |
| --- | --- | --- | --- | --- | --- | --- | --- | --- | --- | --- | --- | --- | --- | --- | --- |
|  | n | IRR (95%CI) | | p | n | IRR (95%CI) | p | n | | IRR (95%CI) | | p | n | IRR (95%CI) | p |
| **Type 1 DM** |  |  | |  |  |  |  |  |  | |  | |  |  |  |
| Unadjusted | 2597 | - |  | | 159999 | 2.28  (1.68,3.10) | <0.001 | 204017 | 3.01  (2.24,4.05) | | <0.001 | | 108922 | 3.97  (2.83,5.58) | <0.001 |
| Model 1 | 2379 | - |  | | 150847 | 2.50  (1.84,3.40) | <0.001 | 190387 | 2.95  (2.15,4.05) | | <0.001 | | 98935 | 3.82  (2.67,5.47) | <0.001 |
| **Type 2 DM** |  |  |  | |  |  |  |  |  | |  | |  |  |  |
| Unadjusted | 2609 | 0.96  (0.14,6.45) | 0.96 | | 161352 | 1.54 (1.18,2.02) | 0.001 | 210291 | 1.48  (1.27,1.74) | | <0.001 | | 119860 | 1.48  (1.33,1.66) | <0.001 |
| Model 1 | 2389 | 1.05  (0.18,6.15) | 0.96 | | 152018 | 1.06 (0.81,1.38) | 0.68 | 195947 | 1.24  (1.04,1.48) | | 0.02 | | 108523 | 1.24  (1.10,1.39) | <0.001 |

**Supplementary Table 4: Number of microvascular complications of diabetes and incident fractures (n = 19,754)**

|  | Diabetes with 1 complication vs diabetes without complications | | | Diabetes with 2+ complications vs diabetes without complications | | |
| --- | --- | --- | --- | --- | --- | --- |
|  |  | IRR (95%CI) | p |  | IRR (95%CI) | p |
| Osteoporotic fracture | |  |  |  |  |  |
| *Unadjusted* |  | 2.08 (1.69,2.56) | <0.001 |  | 4.20 (3.16,5.59) | <0.001 |
| *Model 1* |  | 1.97 (1.57,2.47) | <0.001 |  | 3.69 (2.78,4.91) | <0.001 |

Diabetic complications: Any of diabetic mononeuropathy, polyneuropathy, cataract, retinopathy, arthropathy, glomerular disorders.

Model 1, covariates: sex, age, tobacco use, alcohol use, deprivation index score, level of physical activity, co-morbidities

**Supplementary Table 5: Numbers of participants with each type of complication, by BMI category (row percentages)**

|  | <18.5 kg/m^2^ (Underweight) | 18.5-25 kg/m^2^ (Normal) | 25-30 kg/m^2^ (Overweight) | >30 kg/m^2^ (Obese) |
| --- | --- | --- | --- | --- |
| Eye complications | 4 (0.2) | 327 (13.1) | 887 (35.5) | 1278 (51.2) |
| Neuropathy | 1 (0.1) | 111 (12.0) | 285 (30.7) | 532 (57.3) |
| Glomerular disorder | 2 (0.4) | 62 (13.5) | 139 (30.4) | 255 (55.7) |
| Arthropathy | - | 1 (4.8) | 5 (23.8) | 15 (71.4) |

**Supplementary Table 6: Time since diagnosis of Type 2 diabetes and risk of fracture by one year increment**

| Duration of disease (since diagnosis) | IRR (95%Ci) | p |
| --- | --- | --- |
| Within 1 year | 1.00 (0.72,1.40) | 0.99 |
| 1+ years | 1.16 (1.05,1.28) | 0.002 |
| Within 2 years | 0.96 (0.78,0.18) | 0.72 |
| 2+ years | 1.18 (1.08,1.32) | 0.001 |
| Within 3 years | 0.95 (0.81,1.12) | 0.55 |
| 3+ years | 1.24 (1.11,1.38) | <0.001 |
| Within 4 years | 0.99 (0.86,1.13) | 0.85 |
| 4+ years | 1.26 (1.12,1.42) | <0.001 |
| Within 5 years | 1.02 (0.89,1.17) | 0.77 |
| 5+ years | 1.28 (1.13,1.44) | <0.001 |
| Within 6 years | 1.07 (0.94,1.21) | 0.31 |
| 6+ years | 1.26 (1.10,1.45) | 0.001 |
| Within 7 years | 1.09 (0.97,1.22) | 1.14 |
| 7+ years | 1.26 (1.08,1.46) | 0.002 |
| Within 8 years | 1.12 (1.00,1.25) | 0.05 |
| 8+ years | 1.22 (1.04,1.45) | 0.02 |
| Within 9 years | 1.12 (1.00,1.25) | 0.05 |
| 9+ years | 1.24 (1.04,1.48) | 0.01 |

**Supplementary Table 7: Association between Type 1 and Type 2 diabetes and incident fractures, with the exclusion of individuals with assumptions on age at diagnosis or duration of disease**

|  | Type 1 diabetes vs no diabetes | | | Type 2 diabetes vs no diabetes | | |
| --- | --- | --- | --- | --- | --- | --- |
|  | n | IRR (95%CI) | p | n | IRR (95%CI) | p |
| **Incident osteoporotic fracture** | | |  |  |  |  |
| *Unadjusted* | 402771 | 2.90 (2.34,3.59) | <0.001 | 417242 | 1.37 (1.25,1.51) | <0.001 |
| *Model 1* | 402771 | 3.04 (2.47,3.76) | <0.001 | 417242 | 1.15 (1.04,1.27) | 0.004 |
| *Model 1+eBMD* | 402771 | 2.84 (2.30,3.51) | <0.001 | 417242 | 1.24 (1.13,1.37) | <0.001 |
| *Model 1+fat mass* | 402771 | 3.05 (2.47,3.76) | <0.001 | 417242 | 1.20 (1.09,1.32) | <0.001 |
| *Model 1+CRP* | 402771 | 3.01 (2.44,3.72) | <0.001 | 417242 | 1.15 (1.04,1.26) | 0.006 |
| *Model 1+eGFR* | 402771 | 3.08 (2.49,3.80) | <0.001 | 417242 | 1.15 (1.04,1.26) | 0.005 |
| *Model 2* | 402771 | 2.82 (2.28,3.48) | <0.001 | 417242 | 1.26 (1.15,1.39) | <0.001 |

**Supplementary Table 8: Association between Type 1 and Type 2 diabetes and incident fractures, with the inclusion of individuals with “Possible type 2 diabetes”**

|  | Type 1 diabetes vs no diabetes | | | Type 2 diabetes vs no diabetes | | |
| --- | --- | --- | --- | --- | --- | --- |
|  | n | IRR (95%CI) | p | n | IRR (95%CI) | p |
| **Incident osteoporotic fracture** | | |  |  |  |  |
| *Unadjusted* | 402833 | 3.04 (2.46,3.76) | <0.001 | 419796 | 1.53 (1.39,1.68) | <0.001 |
| *Model 1* | 402833 | 3.16 (2.56,3.89) | <0.001 | 419796 | 1.27 (1.15,1.39) | <0.001 |
| *Model 1+eBMD* | 402833 | 2.95 (2.39,3.64) | <0.001 | 419796 | 1.36 (1.24,1.49) | <0.001 |
| *Model 1+fat mass* | 402833 | 3.16 (2.56,3.90) | <0.001 | 419796 | 1.32 (1.20,1.46) | <0.001 |
| *Model 1+CRP* | 402833 | 3.12 (2.53,3.85) | <0.001 | 419796 | 1.26 (1.15,1.38) | <0.001 |
| *Model 1+GFR* | 402833 | 3.18 (2.58,3.93) | <0.001 | 419796 | 1.27 (1.15,1.39) | <0.001 |
| *Model 2* | 402833 | 2.93 (2.37,3.62) | <0.001 | 419796 | 1.38 (1.26,1.53) | <0.001 |

**Supplementary Table 9: ICD 9 and ICD 10 codes used to identify incident osteoporotic fractures through linkage with hospital episode statistics (HES)**

| **ICD 9** | **ICD 10** | **Osteoporotic fracture** |
| --- | --- | --- |
| 7331 | M80.0 | x |
|  | M80.00 | X |
|  | M80.02 | X |
|  | M80.03 | X |
|  | M80.05 | X |
|  | M80.06 | X |
|  | M80.07 | X |
|  | M80.08 | X |
|  | M80.09 | X |
| 7331 | M80.1 | X |
|  | M80.19 | X |
| 7331 | M80.2 | X |
|  | M80.25 | X |
| 7331 | M80.3 | X |
| 7331 | M80.4 | X |
|  | M80.40 | X |
|  | M80.45 | X |
|  | M80.48 | X |
|  | M80.49 | X |
| 7331 | M80.5 | X |
|  | M80.50 | X |
|  | M80.55 | X |
|  | M80.57 | X |
|  | M80.58 | X |
|  | M80.59 | X |
|  | M80.8 | X |
| 7331 | M80.80 | X |
|  | M80.81 | X |
|  | M80.88 | X |
|  | M80.89 | X |
| 7331 | M80.9 | X |
|  | M80.90 | X |
|  | M80.91 | X |
|  | M80.93 | X |
|  | M80.95 | X |
|  | M80.97 | X |
|  | M80.98 | X |
|  | M80.99 | X |
| 8050, 8051, 8060, 8061 | S12.0 | X |
|  | S12.00 | X |
|  | S12.01 | X |
| 8050, 8051, 8060, 8061 | S12.1 | X |
|  | S12.10 | X |
|  | S12.11 | X |
| 8050, 8051, 8060, 8061 | S12.2 | X |
|  | S12.20 | X |
|  | S12.21 | X |
| 8050, 8051, 8060, 8061 | S12.7 | X |
|  | S12.70 | X |
| 8075, 8076 | S12.8 | X |
|  | S12.80 | X |
| 8050, 8051, 8060, 8061 | S12.9 | X |
|  | S12.90 | X |
| 8052, 8053, 8062, 8063 | S22.0 | X |
|  | S22.00 | X |
|  | S22.01 | X |
| 8052, 8053, 8062, 8063 | S22.1 | X |
|  | S22.10 | X |
|  | S22.11 | X |
| 8072, 8073 | S22.2 | X |
|  | S22.20 | X |
| 8070, 8071 | S22.3 | X |
|  | S22.30 | X |
| 8070, 8071 | S22.4 | X |
|  | S22.40 | X |
| 8074 | S22.5 | X |
|  | S22.50 | X |
| 8090, 8091 | S22.8 | X |
|  | S22.80 | X |
| 8090, 8091 | S22.9 | X |
| 8054, 8055, 8064, 8065 | S32.0 | X |
|  | S32.00 | X |
|  | S32.01 | X |
| 8056, 8057, 8066, 8067 | S32.1 | X |
|  | S32.10 | X |
| 8056, 8057, 8066, 8067 | S32.2 | X |
|  | S32.20 | X |
|  | S32.3 | X |
| 8084, 8085 | S32.30 | X |
| 8080, 8081 | S32.4 | X |
|  | S32.40 | X |
|  | S32.41 | X |
| 8082, 8083, 8088, 8089 | S32.5 | X |
|  | S32.50 | X |
| 8054, 8055, 8064, 8065, 8084, 8085, 8088, 8089, 8090, 8091 | S32.7 | X |
|  | S32.70 | X |
|  | S32.71 | X |
| 8054, 8055, 8064, 8065, 8084, 8085, 8088, 8089 | S32.8 | X |
|  | S32.80 | X |
|  | S32.81 | X |
| 8100, 8101 | S42.0 | X |
|  | S42.00 | X |
|  | S42.01 | X |
| 8110, 8111 | S42.1 | X |
|  | S42.10 | X |
|  | S42.11 | X |
| 8120, 8121 | S42.2 | X |
|  | S42.20 | X |
|  | S42.21 | X |
| 8122, 8123 | S42.3 | X |
|  | S42.30 | X |
|  | S42.31 | X |
| 8124, 8125 | S42.4 | X |
|  | S42.40 | X |
|  | S42.41 | X |
| 8180, 8181 | S42.7 | X |
|  | S42.70 | X |
|  | S42.71 | X |
| 8120, 8121 | S42.8 | X |
|  | S42.80 | X |
| 8120, 8121 | S42.9 | X |
|  | S42.90 | X |
|  | S42.91 | X |
| 8124, 8125, 8130, 8131 | S52.0 | X |
|  | S52.00 | X |
|  | S52.01 | X |
| 8130, 8131 | S52.1 | X |
|  | S52.10 | X |
|  | S52.11 | X |
| 8132, 8133 | S52.2 | X |
|  | S52.20 | X |
|  | S52.21 | X |
| 8132, 8133 | S52.3 | X |
|  | S52.30 | X |
|  | S52.31 | X |
| 8132, 8133 | S52.4 | X |
|  | S52.40 | X |
|  | S52.41 | X |
| 8134, 8135 | S52.5 | X |
|  | S52.50 | X |
|  | S52.51 | X |
| 8134, 8135 | S52.6 | X |
|  | S52.60 | X |
|  | S52.61 | X |
| 8130, 8131 | S52.7 | X |
|  | S52.70 | X |
|  | S52.71 | X |
| 8130, 8131, 8134, 8135, 8180, 8181 | S52.8 | X |
|  | S52.80 | X |
|  | S52.81 | X |
| 8130, 8131 | S52.9 | X |
|  | S52.90 | X |
|  | S52.91 | X |
| 8200, 8201, 8208, 8209 | S72.0 | X |
|  | S72.00 | X |
|  | S72.01 | X |
| 8202, 8203 | S72.1 | X |
|  | S72.10 | X |
|  | S72.11 | X |
| 8202, 8203 | S72.2 | X |
|  | S72.20 | X |
|  | S72.21 | X |
| 8210, 8211 | S72.3 | X |
|  | S72.30 | X |
|  | S72.31 | X |
| 8212, 8213 | S72.4 | X |
|  | S72.40 | X |
|  | S72.41 | X |
| 8210, 8211 | S72.7 | X |
|  | S72.70 | X |
|  | S72.71 | X |
| 8210, 8211 | S72.8 | X |
|  | S72.80 | X |
|  | S72.81 | X |
| 8210, 8211 | S72.9 | X |
|  | S72.90 | X |
|  | S72.91 | X |
| 8230, 8231 | S82.1 | For women |
|  | S82.10 | For women |
|  | S82.11 | For women |
| 8230, 8231, 8232, 8233 | S82.2 | For women |
|  | S82.20 | For women |
|  | S82.21 | For women |
| 8248, 8249 | S82.3 | For women |
|  | S82.30 | For women |
|  | S82.31 | For women |
| 8230, 8231 | S82.4 | For women |
|  | S82.40 | For women |
|  | S82.41 | For women |
| 8240, 8241 | S82.5 | For women |
|  | S82.50 | For women |
|  | S82.51 | For women |
| 8242, 8243 | S82.6 | For women |
|  | S82.60 | For women |
|  | S82.61 | For women |
| 8270, 8271 | S82.7 | For women |
|  | S82.70 | For women |
|  | S82.71 | For women |
| 8244, 8245, 8246, 8247, 8248, 8249 | S82.8 | For women |
|  | S82.80 | For women |
|  | S82.81 | For women |
| 8230, 8231 | S82.9 | For women |
|  | S82.90 | For women |
| 8090, 8091 | T02.1 | X |
| 8058, 8059, 8068, 8069 | T08 | X |
|  | T08.90 | X |

**Supplementary Table 10: formulae for calculation of estimated Glomerular Filtration Rate.**

| ***Sex*** | ***Serum creatinine, SCr (mg/dL)*** | ***Equation (age in years for ≥ 18)*** |
| --- | --- | --- |
| *female* | *≤ 0.7* | *GFR = 142 × (SCr/0.7)^-0.241^ × (0.9938)^Age^ × 1.012* |
| *female* | *> 0.7* | *GFR = 142 × (SCr/0.7)^-1.200^ × (0.9938)^Age^ × 1.012* |
| *male* | *≤ 0.9* | *GFR = 142 × (SCr/0.9)^-0.302^ × (0.9938)^Age^* |
| *male* | *> 0.9* | *GFR = 142 × (SCr/0.9)^-1.200^ × (0.9938)^Age^* |
